# Ageism and Perceptions of Vulnerability: Framing of Age During the COVID-19 Pandemic

**DOI:** 10.1093/geroni/igab046.516

**Published:** 2021-12-17

**Authors:** Jasmin Tahmaseb McConatha, jordan Broussard, Jacki magnerelli

**Affiliations:** West Chester University, West Chester, Pennsylvania, United States

## Abstract

Media representations of the Covid-19 pandemic and its devastating consequences have shaped people’s fears, anxiety, and perceptions of vulnerability. Social scientists have examined the consequences of how information is “framed.” Framing theory asserts that issues can be portrayed differently by emphasizing or de-emphasizing aspects and information. According to Lakoff (2004) the impact of a message is not based on what is said but how it is said. Theories of framing focus on how the media frames issues, which then structure and shape attitudes and policies. A news article serves as a frame for an intended message. The purpose of this project is to analyze the ways that “age” has been framed during the Covid-19 pandemic. One of the most dominant frames in terms of COVID-19 coverage is how the pandemic has been analyzed through the lens of age and framed in terms of age discrimination. Method: A thematic analysis of New York Times and Washington Post news articles addressing older adults and illness vulnerability was conducted. The results of news articles appearing in these prominent newspapers indicated that the perceptions of older men and women tended to focus on the relationship between age and vulnerabilities to severe consequences from Covid-19. The frames in which these new articles were presented indicated ageist tones and messages that had the potential to either reinforce or lead to age stereotyping and discrimination.

